# Extra Supplement—The Nursing Mirror

**Published:** 1890-04-12

**Authors:** 


*The Hospital*, April 12, 1890. *Extra Supplement.*

### "The Hospital" Nursing Mirror.

Being the Extra Nursing Supplement of "The Hospital" Newspaper.

All Contributions for this Supplement should be addressed to the Editor, The Hospital, 140, Strand, London, W.C., and should have the word "Nursing" plainly written in left-hand top corner of the envelope.

### En Passant.

**A DRAMATIC ENTERTAINMENT.**—The Silurian Amateur Dramatic Club and friends have most kindly undertaken to give a performance at the Queen's Gate Hall, on the evening of Monday, April 28th, at eight o'clock, in aid of the funds of the Trained Nurses' Club, 12, Buckingham Street, Strand, under the patronage of many distinguished people. Prices of admission: Stalls, 5s.; reserved seats, 2s. 6d.; unreserved, 1s. Tickets can be had from Mrs. Nichol, at the Nurses' Club. Queen's Gate Hall is in Harrington Road, close to South Kensington Station.

**HAGGERSTON DISTRICT HOME.**—The scheme for district nursing in Haggerston and Hoxton is now fully matured, and an excellent committee, with the Rev. E. P. Pownall for chairman, has been formed. The prospectus makes no mention of the East London Nursing Society, and rather leads its readers to believe that no district nursing is done in the East End. This is a pity; we hope the newcomers will be careful to in no way interfere with the older society, which has been in existence so many years, and has borne the burden and heat of the day.

**BRADFORD NEWS.**—There seems to be an epidemic of mis-understandings amongst Bradford medical men, and both Dr. Hime and Dr. Goyder have been afflicted. Dr. Hime laboured under the belief that the private nurses of the Bradford Institute were maintained by charitable subscriptions, whereas the fact is all the subscriptions and part of the earnings of the private nurses go to support the district nurses. Dr. Goyder proves Dr. Hime in the wrong, and then proceeds himself to express disapproval of a proposed Home for Cancer cases, on the ground that cancer is infectious and is best left to the treatment of the Poor Law surgeon and the district nurse.

**BRISTOL AND CLIFTON INSTITUTION.**—The annual report is not quite so satisfactory as usual as regards funds, but as regards work done it is excellent. The number of nurses on the staff has again been brought up to what it was in the end of 1888, that is 38. Seven pupils were in training at the close of 1889. During the year the nurses did 1,270 weeks' work, taking 350 cases. Besides these the outside staff of nurses, now numbering about 20, had 262 weeks' work, with 100 cases. Seven pupils were received and four nurses left during the year. The Bristol Infirmary Committee towards the close of 1889 intimated their intention of withdrawing from the agreement of many years' standing, whereby the institution pupils had been trained at the infirmary. Consequently other arrangements had to be made, and the institution pupils will now be trained at the Crumpsall Infirmary, Manchester, and University College Hospital, London. The committee have decided to give the nurses a certain amount of uniform dress instead of the clothing allowance which has been given hitherto. The adoption of a uniform has now become so general in nursing establishments that it is thought best to fall in with the practice, which has the advantage of securing a neat and respectable appearance on the part of the nurses, whether at work or out of doors. During the past year arrangements have been made with the promoters of the National Pension Fund for Nurses for the admission of nurses; five have joined. The institution gives £2 a year towards each nurse's subscription so long as she remains in the institution.

**THOUSANDS OF POUNDS FOR THE NATIONAL PENSION FUND.**—We have the pleasure to acknowledge the following sums towards the £14,000 now being raised to at once bring the Donation Bonus Fund up to £40,000. We hope to be able to acknowledge contributions amounting to £1,000 every week until the £40,000 are completed. Contributions of any amount may be sent to the Editor of The Hospital, or to the Manager, National Pension Fund for Nurses, 8, King Street, Cheapside, London, E.C.:

Twelfth Thousand.

| | | | | | | | |
|---|---|---|---|---|---|---|---|
| Mr. W. H. Burns | £500 | 0 | 0 | Messrs. Bompas, Bischoff, Dodgson, and Cox | £50 | 0 | 0 |
| Messrs. Glyn, Mills, Currie, and Co. | 300 | 0 | 0 | Mr. Norman Watney | 25 | 0 | 0 |
| Messrs. Newgass and Co. | 100 | 0 | 0 | Messrs. Price and Pott | 26 | 5 | 0 |

Amount already received **Twelve Thousand Pounds.**

**LADY ROBERTS' HOMES.**—Colonel Gildea, of 7, Knaresborough Place, publishes the following extract from a letter lately received from Lady Roberts: "I am badly in need of funds at present, for I have been supplying eight lady nurses to different military hospitals since October last at a cost of more than 1,000 rupees a month, a drain on my resources I never contemplated; but as the India Office have hitherto refused to allow the Government of India to give any increase to the nursing sisters—so badly needed and so earnestly desired—I have tried as far as I could to supply the want in those places where the fearful increase of enteric fever has made the presence of skilled nurses a necessity. My 'Homes in the Hills' and 'Officers' Hospitals' are now about to open, and there is always a good deal of expense in starting them, repairs after the winter snow, &c., having to be done. In addition to my other work, I am building a home at Quetta, where the garrison is now very large, and where the doctors are crying out for nurses, which I am going to try and supply when the home for them is ready, so I shall be very grateful if you can collect some money for me in England. The parents of a poor young Engineer officer, who died of enteric fever last year at Quetta, are giving me £250 towards this latter undertaking."

**BRADFORD NURSES' INSTITUTION.**—The annual meeting was held lately. The Committee, in presenting their report (which was read by Dr. Dunlop) on the work of the institution for the past year, stated that during the period named 52 nurses and probationers had been engaged, the average staff engaged in private and district nursing being about 35 in number. During the summer and autumn the calls for the services of the nurses became so numerous that it was impossible to meet them, and at one committee meeting no less than 30 telegrams containing applications for nurses, which had to be declined, were laid upon the table. During the year the conduct and efficiency of the nurses had been generally satisfactory. No complaints of a serious nature had required attention. The diminution of nine members of the staff during the year had been counterbalanced by the engagement of new probationers, so that the total on December 31st, 1889, stood at 46 nurses and probationers. Regarding the district or charitable nursing branch, the report stated that the three districts situated in the most densely populated and poorest parts of the town had been faithfully attended to by their separate nurses during the past year. The services thus rendered to the sick poor were not only gratefully acknowledged, but increasingly sought for.

### The Late Mr. Junius S. Morgan.

We have to record with deep and sincere sorrow the death from a carriage accident of Mr. Junius S. Morgan. Neither the readers of this journal nor the public at large have any idea of the high but modest worth, and the generous liberality of Mr. Morgan's character and life. It adds to the sorrow we feel at the loss of him when we think of his sudden and painful death. Mr. Morgan has lived during the winter for several years at Monte Carlo. On Thursday week he was driving in his Victoria from Beaulieu, and when near the village of Eze, where the road runs parallel with the railway, a train rushing past frightened the horses, and caused them to bolt at a furious speed. Mr. Morgan was thrown out of the carriage, and the coachman did not miss him until the horses had galloped for a considerable distance. On returning, he found his master lying insensible on the road. He was taken home, and Dr. Pickering was sent for, who found Mr. Morgan suffering from concussion of the brain. Besides this, the unfortunate gentleman was wounded in the forehead, his lips were split, his nose severely injured, and the bones of his left hand broken. So terrible, indeed, were the injuries that he never recovered consciousness, and died on Tuesday last.

Mr. Junius Morgan was one of those men whom all right-minded people loved to honour. At the time of his death he was a great merchant, who had been trusted and admired for many years by the sternest judges of character that can be found anywhere in the modern world. This great merchant prince was not, however, born to greatness; he achieved greatness for himself. In youth he knew what poverty, or if not poverty, at least what straitened means meant. Sixty-six years ago—for he was seventy-seven at the time of his death—he was a youth in a dry goods store. In the firm in which he began his business career he rose to be a partner. That firm had dealings with the late Mr. George Peabody. Mr. Peabody soon discovered the character of Mr. Morgan, with the result that in 1855 the latter was offered a partnership in the famous firm of George Peabody and Co. Ten years later Mr. Peabody retired from business, and Mr. Morgan became head of the firm, which was thenceforth, and until the time of his death, known as J. S. Morgan and Co.

In the City of London, where the business of J. S. Morgan and Co. was principally carried on, Mr. Morgan occupied a peculiar and almost unique position. Physically large and massive, his intellect was also of the strong and striking order. He was a man who made money, but he made it by steady and persistent attention to business, and by fair and straightforward dealing. He secured a reputation by honestly earning it, and he maintained it by the same means. That reputation thus secured and maintained, brought fortune to his house, and kept her there. But Mr. Morgan was not merely a great and successful merchant; he was a bright, kindly, lovable, and generous man. The conductors of this journal, and still more the responsible heads of the National Pension Fund for Trained Nurses, knew better than most men the depth of feeling, and the large liberality of his mind, towards hospital nurses, and all those who were charged with the care of the sick. From the very first moment when his attention was called to the hard lives and poor rewards of the nurses, Mr. Morgan took a deep and active interest in the improvement of their condition. When the scheme for the establishment of a National Pension Fund was first brought to his notice he recognised its value immediately. With characteristic promptitude he at once offered to contribute £5,000 towards the twenty thousand needed to establish the fund securely, if others could be found to complete the full amount. But he did more than this; for though he wished as many as possible to be associated with him in order that the movement might have a wide and strong basis of support, he intimated distinctly that if necessity arose he would contribute the whole £20,000 himself.

Mr. Morgan, notwithstanding his princely generosity, had a great dread of diminishing the self-respect and impairing the energy of self-help in those whom he wished to benefit. Having passed successfully through a heroic struggle himself, and come out the better for it, he knew the value of self-reliance and of brave and steady effort both to men and women alike. Therefore, whilst resolved to better the condition of trained nurses, he was minded to do it in the best of all ways, by helping them to help themselves. Hating the idea of pauperism for himself, he knew that to every right-minded nurse pauperism must be as hateful as it was to him. When, therefore, a self-helping scheme for nurses was brought to his notice he placed himself heart, soul, and purse in the vanguard of the movement.

Mr. Morgan, as has been said, gave the first £5,000 to the Pension Fund. Later on he contributed a second £5,000, and was prepared, if necessity arose, to turn his contribution of £10,000 inio £20,000. Last October Mr. Morgan wrote: "I rejoice most heartily at what has been accomplished, and I look forward with the fullest confidence to the future. I approve fully what is proposed in respect to the Bonus Fund" (*i.e.*, to increase it to £40,000), "but we should not rest until that is made up to £50,000, and I am sure that we shall get it." But this by no means represented all that he did for nurses. The managers of the Pension Fund have had many tales of poverty and sorrow poured into their ears. It is not at all a rare occurrence for a woman to be found who, after devoting all her life to the care of the sick, finds herself without either money or home as years advance. Even within the brief period of the life of the National Pension Fund, at least half-a-dozen such destitute and homeless nurses have come to its doors as the last resort of their despair. Mr. Morgan was instinctively turned to by the managers, with assured confidence. In every case immediate help was given, and where necessary, permanent pensions were provided. Nobody knew of these things except the managers of the Fund. Mr. Morgan would not permit a single word to be made public on the subject.

It is known to a good many that her Royal Highness the Princess of Wales is to give special certificates to the first thousand nurses who joined the fund. The managers desired that the ceremony should take place at Roehampton, and that Mr. Morgan should himself have the honour of receiving her Royal Highness at his residence. But with characteristic modesty Mr. Morgan quietly but firmly put aside the suggestion. He was willing to give of his substance, but he was not willing that his giving should be known.

The National Pension Fund was but one of many institutions which shared in Mr. Morgan's liberality. Quite recently he gave the splendid donation of £10,000 to augment the failing resources of Guy's Hospital. So well known, indeed, notwithstanding his modesty, were his good works that he was inundated with begging letters of every conceivable kind. It must not, however, be supposed that Mr. Morgan was careless in his liberality. On the contrary, he made it his business constantly to consult those who had better knowledge than himself, and on whose judgment he could rely. How much the various charities have received at his hands will never be known, for Mr. Morgan gave privately and upon a definite principle known only to himself. His views, however, are well expressed in a letter he wrote to us on the eve of his departure for Monte Carlo, and we commend his words to the consideration of everybody, and especially to the prosperous of all classes. "As regards the little that I am able to do in the way of assistance to others, who are less fortunate than myself, I can assure you no human being gets one half the happiness that I do out of it. . . . I don't believe there is a truer saying in Holy Writ than that 'It is more blessed to give than to receive,' and happy is the man that learns it." A remarkable confession by a remarkable man, which will no doubt lead many to try so simple a prescription for securing the highest form of happiness this world can afford.

Such was Mr. Junius Morgan, and such were some of his many good deeds. He has passed away, and the sick nurses of this country, as well as the charitable institutions, have lost a noble and sympathetic friend. What can we do to honour the memory of so just, so wise, so good a man? We have recorded our sense of his worth; what can the nurses do towards whom his heart was so warm? And what can the charities do which he has so often and so liberally helped? It does not as yet occur to us what might be fit for the occasion, but we feel that deeds, and not words alone, should show to Mr. Morgan's family that he is honoured in his death as in his life.

### Everybody's Opinion.

[*Correspondence on all subjects is invited, but we cannot in any way be responsible for the opinions expressed by our correspondents. No communications can be entertained if the name and address of the correspondent is not given, or unless one side of the paper only be written on.*]

### THE SOUTHSEA HOME.

General Ritchie writes: Noticing in your number of 22nd a comparison between the Southsea Home for Sick Children and that at St. Anne's-on-Sea as to cost of maintenance, I should like to inquire how many beds there are in the latter? Everything depends upon the time the patients are retained in the home when making comparisons. Here children are often kept for months. The number of beds (if always occupied) should be the means of comparison, not the number of patients passing through the home in the year. We have 18 beds, and the cost per bed, as you can easily calculate, is £494, which, divided by 18, is equal to £27 nearly. I do not know what the cost per bed is at St. Anne's-on-Sea, but I do not think £27 is much for food, lodging, medical and surgical necessaries at a place like this, where rent and wages are very high.

### UNIFORMS FOR JUNE.

One Interested writes: I should be very glad of any information respecting the dress to be worn on the day our gracious Princess presents us with our certificates. You say all the nurses are busy preparing pretty dresses. I should be glad of any nurse's opinion who, like myself, belongs to no institution, but works for herself, therefore has no uniform. Would you kindly give this letter publication, so that nurses not in uniform could suggest a pretty dress for the occasion?

### Appointments.

[It is requested that successful candidates will send a copy of their applications and testimonials, with date of election, to The Editor, The Lodge, Porchester Square, W.]

London Hospital.—Miss Marion Wilson has been appointed Sister of the Queen Victoria wards, and Miss Janet Hamilton has been appointed night sister. Both were trained at the London Hospital.

Whitechapel Infirmary.—Miss Coghlan, late lady superintendent of the Fleming Memorial Hospital, has been appointed matron of this infirmary, in place of Mrs. Perry, who resigns after many years of hard work, during which she inaugurated trained nursing.

Royal Albert Edward Infirmary, Wigan.—Miss K. V. Macintyre was on April 5th appointed matron of this infirmary out of a large number of candidates. For the last two years Miss Macintyre has acted as matron of the Carmarthen Infirmary, to which post she was appointed after a year's work as sister at the National Hospital in Queen's Square. For five years Miss Macintyre acted as sister at the London, and she has also worked at Bradford Fever Hospital and Glasgow Royal Infirmary. It is, therefore, evident that Miss Macintyre takes great and wide experience to her new post, and her excellent testimonials prove that she is a capable manager and a general favourite.

Clayton Hospital, Wakefield.—Miss J. Johnstone Hunter was last week elected matron of this hospital. Miss Hunter trained at Guy's, and was for two years matron of the Scarboro' Hospital, and for the last three years matron of the Lowestoft Hospital. For a short time, also, Miss Hunter had charge of Professor Jessop's private hospital at Leeds, and her testimonials from all those for whom she worked are of the highest order.

### Love.

My love has been spurned and thrust aside,
My heart is wounded and sore;
Then a Voice said softly, "Love on still
With stronger love than before."

But this aching loss, this weary pain,
They are more than I can bear;
"Love on," said the Voice, "for only love
Can save you from blank despair."

But this hurt has come from one whose life
I had only longed to bless;
It is bitter pain! "Love," said the Voice—
"Love will heal all bitterness."

But can I forgive when life is changed,
And my trust and hope have gone?
"Love and you must forgive," said the Voice—
"Love only, love on, love on!"

But the hurt remains, and the help I had
From that other heart has fled;
"Love with no hope of return. Love on,"
Said the Voice, "till self is dead."

But may I hope for no joy to come
From the love that I have given?
"Love on through life and death," said the Voice,
"Love in itself is heaven."

### Presentations.

Lambeth Infirmary.—The first anniversary of the nurses' working meeting was held in the matron's drawing-room of the Lambeth Infirmary on the 24th ult. At the matron's request the chaplain presented a brief report of the year's work, which showed most encouraging results. The work meetings are held every Monday evening. Upwards of £12 has been made by the sale of work. The profits of this labour of love have been applied to useful and deserving objects. The meeting was also made to serve another purpose than the announcement of its success, namely, the occasion of rendering a well-deserved tribute to one of the lady officers of the infirmary. For some time past the nurses had determined to present Miss Tomkins, the assistant matron, with a testimonial. This purpose arose entirely within their own circle, and owed nothing to any suggestions from without. Miss Tomkins has held her present position for nearly 11 years, and by her kind and gentle bearing has won the respect and attachment of all who have come into official relations with her. The testimonial consisted of an electro-plate basket, with the monogram engraved in the centre. It was elegant in design, and was greatly admired. The chaplain presented this gift in the name of the nurses, and in the course of his remarks dwelt upon those noble and winning qualities of Miss Tomkins' character which have made her such a favourite with the nurses, and won for her that admiration which he well knew to be most real and sincere. Miss Tomkins, who was much surprised and touched by the unexpected honour, briefly acknowledged the present. Afterwards, Miss Griffiths kindly invited the company to supper. This was followed by a musical entertainment, and thus this most pleasant meeting was brought to a close.

Mr. J. J. Weaver has been presented with a handsome clock by the nurses of Southport Infirmary, on the occasion of his resigning his connection with that institution.

### Lectures.

The Gresham lectures on "Physic" will be delivered at six p.m., at Gresham College, on April 15th, 16th, 17th, and 18th. Admission free.

The Ancient Merchants' Lectures on "Social Problems" will be delivered during April by the Rev. Alfred Rowland, at the Memorial Hall. Admission free.

The next lecture at the Midwives Institute and Trained Nurses' Club will be given at 12, Buckingham Street, on Friday evening, April 18th, at a quarter to eight, by Dr. Tom Robinson. Subject: "Hypnotism."

### Notes and Queries.

To Correspondents.—1. Questions may be written on post-cards. 2 Advertisements in disguise are inadmissible. 3. In answering a query please quote the number. 4. If a private answer is desired, a stamped addressed envelope must be forwarded.

Notice.—We must really ask our correspondents to be more particular in enclosing name and address. Constantly we receive queries or letters with no name attached.

### Queries.

(1) *Training without Premium.*—Where can I obtain a year's training as nurse without paying a premium?—*Kate.*

### Answers.

(71) *American Hospitals.*—There is no list published in England. The State Charities Aid Association of New York have published one, and Putnams have published a hand-book on American "Training Schools for Nurses."

(73) *Pharmacy.*—Can be learnt on equal terms with men. The Pharmaceutical Society, 17, Bloomsbury Square, charges £4 4s. for a course of instruction; the South London School in Kennington Road, charges £15 for one year's training.

(76) *Caps.*—Would not your best plan be to go and see the nurse-dolls and copy your caps from one seen there?

*Nurse B.*—(1) Use better suspenders; (2) The only term is "laying out the dead."

*Easter Wishes.*—Many thanks to our kind friends who have sent us Easter greetings; we are specially grateful for an elaborate card from Hastings.

### Vacancies.

The following vacancies are announced:

### Matrons.

Glasgow Deaf and Dumb Institution; £80.

Kensington Infirmary (assistant); £40.

### Nurses.

Abbey Poorhouse, Paisley; £25.
Aimee Nursing Homes.
Blackheath Nursing Institute.
Bournemouth Victoria Home; £25.
Blackburn Infirmary; £22.
Brixton and South London Institute.
Birkenhead Borough Hospital; £25.
Coventry Hospital; £20.
Cardiff Infirmary; £24.
Carmarthen Infirmary; £23.
Croydon Hospital.
Cheltenham Hospital.
Chelsea Infirmary; £18.
Dumfries Infirmary (Fever); £20.
Devon and Exeter Hospital (Superintendent); £60.
Edinburgh Poorhouse; £25.
Guildford Hospital; £20.
General Nursing Institute.
Hanwell Ophthalmic School; £18.
Home for Incurables, Westbourne Park.
Hull Nurses' Home.
Herts General Infirmary (night); £12.
Ipswich Nurses' Home.
Jessop Hospital, Sheffield; £25.
Kingston Home, Hull.
King's Cross Hospital, Dundee; £27.
Lowestoft Hospital (night); £20.
Liverpool Stanley Hospital; £25.
Miss Stewart's Institute, Glasgow.
Mr. Wilson's Institution.
Norwich Union; £25.
Nursing Institute, Newport, I.W.
North-West London Hospital (night); £24.
Newcastle-on-Tyne Royal Infirmary; £20.
Peterboro' Infirmary.
Royal Hospital for Children and Women.
Rivers Street Home, Bath.
Stamford Infirmary; £25.
Stockport Infirmary.
St. Anne's School, Redhill; £30.
South-Eastern Fever Hospital; £30.
Stephen Monckton Nurses' Home.
The Coppice, Nottingham (attendant).
Victoria Hospital for Children, Hull; £20.
Workhouse Infirmary Nursing Association.
Wonford House, Exeter.
West Bromwich Hospital (head); £30.
West Bromwich Hospital (staff); £25.
Wirral Children's Hospital (head).
Whitechapel Infirmary; £20.
Wakefield Hospital; £25.
Wakefield Hospital (night); £20.
Yeovil Hospital; £14.

### District Nurses.

Cambridge Home for Nurses. Matron

Market Drayton (midwife).

Rivers Street Home, Bath.

### Nursery Attendant.

Lewisham Union; £20.

### Probationers.

Coldstream Cottage Hospital.

Halifax Fever Hospital; £12.

Leeds Nurses' Institution; £14.

Notts Nursing Association.

Stoke-on-Trent Nurses' Home.

Tamworth Cottage Hospital.

Workhouse Infirmary Nursing Association.

Wisbech Hospital.

### Amusements and Relaxation.

**N.B.—New Word Competition Commences April 5th, 1890, ends June 28th, 1890.**

Three prizes of 15s., 10s., 5s., will be given for the largest number of words derived from the words set for dissection.

Proper names, abbreviations, foreign words, words of less than four letters, and repetitions are barred; plurals, and past and present participles of verbs, are allowed. Nuttall's *Standard* dictionary only to be used.

N.B.—Word dissections must be sent in WEEKLY, arranged alphabetically, with correct total affixed.

N. The word for dissection for this, the SECOND week of the quarter, being

"REVIEW."

| Names. | Ap. 3rd. | Totals. | Names. | Ap. 3rd. | Totals. |
|---|---|---|---|---|---|
| Holland | 22 | 1077 | Lauriston | | 1004 |
| Red Cape | — | 132 | B. E. T. | — | 453 |
| Nurse Marie | — | 499 | Jacko | — | 110 |
| Garfield | — | 243 | Lady Clara | — | 258 |
| Midget | — | 894 | Patience | 22 | 1131 |
| E. M. S. | — | 149 | Ecila | 23 | 1114 |
| Sister | — | 591 | Maude | — | 690 |
| Duchess | — | 637 | Ruby | — | 698 |
| Ellice | — | 1076 | Esperance | 22 | 1110 |
| Reldas | 22 | 1137 | M. W. | 22 | 1111 |
| Jenny Wren | 22 | 993 | Crystal Palace | — | 698 |
| Mopus | — | 122 | Jackanapes | 17 | 772 |
| Neptune | — | 118 | M. M. M. | — | 25 |
| Qu'appelle | 20 | 1016 | A. F. | — | 318 |
| Whitehoe | — | 880 | E. S. | — | 49 |
| Nurse Clague | — | 40 | | | |

RESULTS OF FOURTH QUARTERLY WORD COMPETITION.

*First Prize.*—15s is awarded to Reldas (Miss Alice Sadler, 32, Norland Square, W.).

*Second Prize.*—10s. is awarded to Patience (Miss F. Calisé, Craven Road, Newbury).

*Third Prize.*—5s. to Ecila (Miss Todd, Moulsor, Newport Pagnell.

### Notice to Correspondents.

N.B.—All letters referring to this page which do not arrive at 140, **Strand, London, W.C.,** by the *first post on Thursdays*, and are not addressed PRIZE EDITOR, will in future be disqualified and disregarded.

